# 5G High-Precision Positioning in GNSS-Denied Environments Using a Positional Encoding-Enhanced Deep Residual Network

**DOI:** 10.3390/s25175578

**Published:** 2025-09-06

**Authors:** Jin-Man Shen, Hua-Min Chen, Hui Li, Shaofu Lin, Shoufeng Wang

**Affiliations:** 1 School of Information Science and Technology, Beijing University of Technology, Beijing 100124, China; shenjinman@emails.bjut.edu.cn (J.-M.S.); lihui@bjut.edu.cn (H.L.); 2 College of Computer Science, Beijing University of Technology, Beijing 100124, China; linshaofu@bjut.edu.cn; 3 AsiaInfo Technologies (China) Inc., Beijing 100094, China

**Keywords:** high-precision positioning, CSI, positional encoding, deep residual network, multi-scale feature extraction, PE-MSRN

## Abstract

With the widespread deployment of 5G technology, high-precision positioning in global navigation satellite system (GNSS)-denied environments is a critical yet challenging task for emerging 5G applications, enabling enhanced spatial resolution, real-time data acquisition, and more accurate geolocation services. Traditional methods relying on single-source measurements like received signal strength information (RSSI) or time of arrival (TOA) often fail in complex multipath conditions. To address this, the positional encoding multi-scale residual network (PE-MSRN) is proposed, a novel deep learning framework that enhances positioning accuracy by deeply mining spatial information from 5G channel state information (CSI). By designing spatial sampling with multigranular data and utilizing multi-source information in 5G CSI, a dataset covering a variety of positioning scenarios is proposed. The core of PE-MSRN is a multi-scale residual network (MSRN) augmented by a positional encoding (PE) mechanism. The positional encoding transforms raw angle of arrival (AOA) data into rich spatial features, which are then mapped into a 2D image, allowing the MSRN to effectively capture both fine-grained local patterns and large-scale spatial dependencies. Subsequently, the PE-MSRN algorithm that integrates ResNet residual networks and multi-scale feature extraction mechanisms is designed and compared with the baseline convolutional neural network (CNN) and other comparison methods. Extensive evaluations across various simulated scenarios, including indoor autonomous driving and smart factory tool tracking, demonstrate the superiority of our approach. Notably, PE-MSRN achieves a positioning accuracy of up to 20 cm, significantly outperforming baseline CNNs and other neural network algorithms in both accuracy and convergence speed, particularly under real measurement conditions with higher SNR and fine-grained grid division. Our work provides a robust and effective solution for developing high-fidelity 5G positioning systems.

## 1. Introduction

With the widespread deployment of 5G networks, high-precision wireless positioning services have become a crucial foundation for a variety of remote sensing applications [1]. 5G positioning provides enhanced spatial resolution and low-latency data acquisition, enabling more timely and precise geospatial analysis. Applications such as environmental monitoring, disaster response, and land use mapping increasingly rely on high positioning accuracy to align remote sensing data with ground truth effectively [2]. In smart healthcare scenarios, real-time patient positioning and trajectory monitoring typically require a positioning error of less than 10 m [3]. In large transportation hubs, vehicle scheduling and management also demand a positioning accuracy within 10 m to ensure operational safety. The intelligent manufacturing sector poses even higher requirements, needing positioning accuracy within 5 m for efficient management of personnel and critical materials [4,5]. In environments such as indoor autonomous driving and logistics management, systems are often expected to achieve sub-meter or even sub-30 cm accuracy to guarantee the safety and real-time obstacle avoidance capabilities of unmanned systems [6].

However, due to multipath interference, non-line-of-sight (NLOS) propagation, and signal obstruction in complex environments, traditional algorithms based on time difference of arrival (TDOA), RSSI, TOA [7,8,9], and other single-type measurement data relying on manually selected features and shallow models are limited in their ability to extract deep spatial relationships from heterogeneous measurement data and thus fail to meet the aforementioned high-precision requirements in practical 5G scenarios [10,11]. To address these challenges, in recent years, 5G positioning algorithms based on deep learning and machine learning have provided new opportunities for improving localization accuracy [12]. By collecting multi-dimensional features at spatially distributed points and integrating multi-source wireless measurements, such as high-dimensional CSI, angle, delay, and the estimation of signal-to-noise ratio (SNR) [13], these methods enable high-precision positioning and demonstrate strong adaptability to complex environments.

Accordingly, the construction of high-quality feature datasets and the design of deep learning algorithms have become hot research topics for enhancing 5G positioning accuracy [14,15,16]. On the one hand, recent studies show that fusing spatial prior information and multi-scale features can significantly enhance the modeling of spatial distributions in complex wireless environments [17,18,19]. On the other hand, the introduction of deep neural networks (such as convolutional and residual networks) enables efficient extraction of complex relationships among high-dimensional features, achieving highly accurate positioning [20,21].

While deep learning has shown promise, a significant research gap remains [17,18,19,20,21]. Many existing models struggle to fully represent the complex spatial characteristics embedded in 5G CSI data. Specifically, they often lack: (1) a mechanism to explicitly encode directional and positional information into the input features, leading to the loss of crucial spatial cues; and (2) a network architecture capable of simultaneously capturing features at multiple scales and depths without suffering from gradient degradation. This limits their accuracy and robustness in challenging real-world environments. To bridge this gap, we propose PE-MSRN, a 5G positioning algorithm based on positional encoding and an image-based deep residual network. Our main contributions are as follows:

- The positional encoding encodes the angle of arrival (AOA) information from the CSI data measured by a 5G uplink positioning signal sounding reference signal (SRS). By extending the encoded feature vectors into 2D images to enhance spatial location expression in 5G CSI, the positioning accuracy is improved. It enables the network to capture spatial relationships between features, improving the interpretation of directional information from the environment. This design enhances the spatial representation and significantly improves positioning accuracy, as detailed in Section 4.2.
- The residual connection network is applied to facilitate the training of deep neural networks and enhance the expression of spatial information by allowing the original input of a residual block to bypass intermediate layers and be directly added to the transformed output of the convolutional block. This design helps to preserve and reinforce original input information, allowing the network to reduce the risk of information degradation with the increase in network depth, build deeper feature representations, alleviate the gradient vanishing problem, and promote faster and more stable convergence during training, as illustrated in Section 4.3.
- The multi-scale feature extraction is introduced by using parallel convolutional branches with different-sized convolutional kernels to process the input features, allowing the network to capture key positional information at different scales and enabling it to detect even subtle feature variations. By incorporating multi-scale information, it enhances the capacity of the network to recognize complex patterns in the data, improving the robustness of its predictions and positioning accuracy, as discussed in Section 4.4.
- In the simulation experiments, the positioning performance of the proposed algorithm is evaluated across different datasets with varying granularities in both 2D and 3D scenarios. Experimental results show that the proposed algorithm exhibits superior convergence stability and outperforms traditional methods in terms of average error and convergence speed. It achieves stable sub-meter and even 20 cm high-precision positioning, meeting the practical accuracy requirements for various scenarios, as shown in Section 5.

The rest of this paper is organized as follows. Section 2 summarizes the related works. Section 3 outlines the overview of the 3D positioning architecture for the deep learning-based 5G uplink positioning system and provides a detailed description of the CSI measurement algorithm utilizing 5G SRS. The detailed description of the proposed algorithm and the analysis of algorithmic complexity are given in Section 4. Section 5 provides the simulation results and analysis, followed by the conclusions being drawn in Section 6. In addition, for ease of understanding, the notation and operation descriptions of this paper are declared in Table 1.

## 2. Related Works

In recent years, with the continuous evolution of 5G communication technology, 5G systems have demonstrated significant advantages in terms of large bandwidth, multiple antennas, and high-dimensional channel measurements, which have brought new development opportunities for wireless positioning technologies [21,22,23]. The 3rd Generation Partnership Project (3GPP) has proposed different accuracy requirements to support typical 5G application scenarios such as emergency rescue, smart healthcare, intelligent manufacturing, autonomous driving, and logistics management. According to 3GPP standards such as TS 22.104 and TS 38.305 [24,25], for public safety and emergency rescue scenarios, user equipment is required to achieve a positioning error of less than 10 m to enable timely target searching and scheduling [26]. In the field of intelligent transportation and vehicle management, including autonomous driving and high-precision mapping, 3GPP requires positioning accuracy to be the sub-meter level, and in certain cases the error must be controlled within 0.5 m [27]. In addition, for intelligent manufacturing, industrial Internet of things (IoT), and smart factories, 3GPP recommends a positioning accuracy of below 30 cm to meet the precise tracking and management needs of equipment, personnel, and assets [28]. These standards set higher requirements for wireless signal processing, network training, and algorithm optimization.

Currently, 5G positioning technologies mainly include traditional methods such as RSSI [29], TOA [30], TDOA [7,8,9], and AOA [31], as well as new methods such as carrier phase positioning [32], enhanced cell identifier (ID), and  multi-round trip time (multi-RTT) positioning [25]. In recent years, multi-source information fusion positioning based on 5G has become a research hotspot, including the integration of 5G with inertial measurement unit (IMU), barometer, and other sensor data, as well as the fusion of multiple 5G measurement values for positioning [33,34,35]. With the introduction of machine learning and deep learning techniques, researchers have conducted extensive research on high-precision positioning in 5G environments and begun to use large-scale data to train neural networks to improve localization accuracy. Deep learning-based 5G positioning approaches have gradually been introduced into the field of wireless localization, resulting in remarkable breakthroughs [14,15,16,17,18]. Most research focuses on the construction of high-quality measurement datasets and the design of neural networks with enhanced positioning performance.

Li et al. proposed a Transformer-based 5G positioning algorithm, in which a rectangular patch strategy was designed to divide channel estimation results into matrices that are then fed into a self-attention-based learning network [21]. Butt et al. proposed a ray tracing-based method that constructs a fingerprint database by measuring reference signal receiving power (RSRP), with a machine learning-assisted 5G positioning scheme and achieving an average positioning error of 1–1.5 m [36]. Fan et al. proposed a structured bidirectional long short-term memory (SBi-LSTM) recurrent neural network (RNN) architecture, where the input features include AoA, time delay, and RSSI, to address the problem of CSI-based three-dimensional terahertz indoor localization [19]. An image enhancement method based on a channel frequency response (CFR) matrix was proposed to construct an image dataset, and the multipath Res-Inception (MPRI) model was designed based on ResNet and Inception architectures [18]. The results verified that the algorithm improves both training speed and positioning accuracy. Suah et al. proposed using synchronization signal reference signal received power (SS-RSRP) and transmitter beam ID data between users and base stations in 5G communications as positioning data, with a deep neural network (DNN)-based model employed for matching, and the method is suitable for small cell environments [37]. Hu et al. integrated 5G and geomagnetic field strength to improve the quality of the dataset and adopted a two-stage long short-term memory (LSTM)-based model for position correction, achieving a positioning accuracy of 0.72 m [38].

In summary, research on deep learning-based 5G positioning has largely focused on multi-source information fusion, the construction of the feature databases suitable for deep network training, and the design of deep neural network architectures to improve positioning accuracy and robustness. Although existing methods have achieved good results in some scenarios, they lack the capability to realize high-dimensional feature image representation of CSI through position encoding-based feature expansion and also lack mechanisms that utilize residual network structures and multi-scale spatial feature extraction to enhance the discriminative ability of a network for spatial structural features.

To address these shortcomings, a deep residual network integrating positional encoding and image-based input is proposed. This algorithm combines residual network structures with multi-scale feature extraction mechanisms and performs systematic research and validation across 3D and 2D application scenarios with different positioning accuracy requirements. Comparative algorithms are also designed to demonstrate its effectiveness. Simulations are conducted on datasets covering three scenarios with varying accuracy requirements: the vehicle flow management in outdoor highway scenarios (accuracy requirement < 10 m), smart factory tool tracking (accuracy requirement < 4 m), and indoor autonomous driving (accuracy requirement < 30 cm) [25,26,27,28]. The experimental results show that the proposed method can achieve positioning accuracies of approximately 2 m, 1.2 m, and 20 cm in the three different scenarios, respectively, meeting the positioning needs of various high-precision applications and demonstrating good practical value.

## 3. Detail Design of CSI Preprocessing

### 3.1. 5G Positioning System Architecture

**Figure 1:**
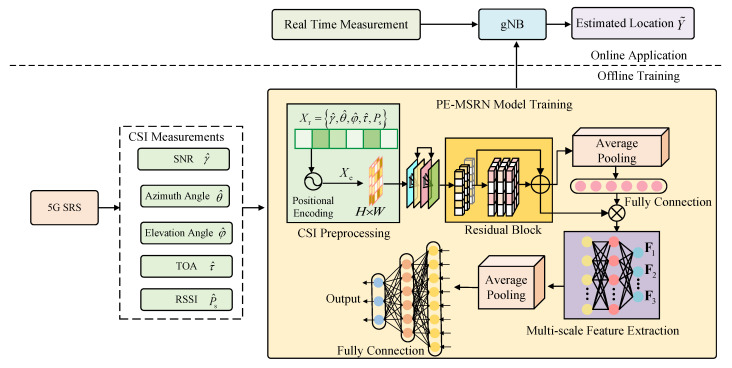
Overview of 3D positioning architecture for deep learning-based 5G uplink localization.

The system architecture of the 5G positioning system presented in this paper is illustrated in Figure 1, which consists of three main stages: CSI preprocessing, offline training, and online application. Firstly, a measurement of the collected SRS data is performed to obtain the CSI feature $X_{r}=\left\{\hat{\gamma },\hat{\theta },\hat{\varphi },\hat{\tau },{\hat{P}}_{s}\right\}$, where $\hat{\gamma }$, $\hat{\theta }$, $\hat{\varphi }$, $\hat{\tau }$, ${\hat{P}}_{s}$ correspond to the estimated SNR, the estimation of azimuth angle and elevation angle, the estimated time delay, and the received signal power. These features undergo data preprocessing, including feature extension and positional encoding enhancement, to improve their representational ability. Secondly, the extended multi-dimensional features $X_{e}$ are converted into an H×W image suitable for deep learning network training.

During the offline training, the system trains multiple deep learning networks using the processed data. Each network has a different architecture and optimization strategy to improve positioning accuracy. Finally, in the online application phase, the trained networks are deployed to the base station to perform positioning predictions using real-time collected CSI data. The system estimates the location of the device based on the measured data and outputs the final positioning result.

The entire system learns the mapping relationship between CSI features and actual coordinates during the offline training phase and performs real-time positioning during the online phase to guarantee the efficiency and 5G positioning accuracy.

### 3.2. Design of CSI Measurement

In the data preprocessing phase, the CSI measurements of the collected SRS obtain $X_{r}=\left\{\hat{\gamma },\hat{\theta },\hat{\varphi },\hat{\tau },{\hat{P}}_{s}\right\}$. The MUSIC algorithm is used for θ and ϕ angle measurement [39,40]. The estimated angles $\left(\hat{\theta },\hat{\phi }\right)$ can be calculated as follows

(1) $$\begin{array}{c} \left(\hat{\theta },\hat{\phi }\right)=\arg\max_{\theta ,\phi }\Gamma \left(\theta ,\phi \right)=\arg\max_{\theta ,\phi }\frac{1}{w^{†}\left(\theta ,\phi \right)U\times U^{†}w\left(\theta ,\phi \right)}, \end{array}$$

where Γ(θ,ϕ) represents the MUSIC spectrum, w(θ,ϕ) is the array response matrix, and U is the matrix of eigenvectors of the noise covariance matrix. The superscript † represents the Hermitian transpose of a matrix.

The signal propagation delay is calculated by using the cubic spline algorithm to obtain the relative distance [40,41]. The cross-correlation-based TOA estimation algorithm is a commonly used delay detection method. The received signal captured by the receiver device from the transmitted signal is subjected to a Fourier transform (FT) in the time domain to obtain the frequency–domain received signal. Then, a least squares (LS) channel estimation is performed [42], providing the estimated channel frequency response (CFR) under the LS criterion, as follows

(2) $$\begin{array}{c} H_{\mathrm{est}}\left(k\right)=H\left(f_{k}\right)+V\left(k\right)S{\left(k\right)}^{†}=\sum_{l=1}^{L-1}\alpha_{l}e^{-j2\pi (f_{c}+k\Delta f)\tau }+V\left(k\right)S{\left(k\right)}^{†}, \end{array}$$

where *k* represents the number of frequency domain sampling points. $S\left(k\right)$ is the original transmitted signal, $f_{c}$ is the center frequency of the OFDM signal, Δf is the frequency domain sampling interval, and V(k) represents the noise. The core task of transmission delay detection is to overlay the power delay profile (PDP) between the received signal and the reference signal [41]. By analyzing the peak position of the power delay profile, the arrival delay of signal can be calculated in units of the Fourier transform time interval, as given by

(3) $$\begin{array}{cc} \hat{\tau }=\frac{i}{M\mu }|i & =\max\left(\mathrm{PDP}\right)=\frac{1}{N_{R}L_{s}}∥\sum_{r=1}^{N_{R}}\sum_{l=1}^{L_{s}}{\left(\mathrm{IFFT}\left(H_{\mathrm{est}}\right)\right)}_{r,l}∥,i\in \left\{1,\ldots ,M\right\}. \end{array}$$

where μ represents the subcarrier spacing and *M* represents the IFFT size of the calculated channel. $N_{R}$ indicates the number of receive antennas, and $L_{s}$ represents the number of allocated signal resources in time domain.

The channel estimation is performed using the LS and minimum mean square error (MMSE) methods, and the SNR is then estimated by comparing the signal power and noise power, as shown in the following

(4) $$\begin{array}{c} \hat{\gamma }=10\times \log_{10}\left(\frac{P_{s}}{P_{n}}\right)=10\times \log_{10}\left(\frac{\sum_{i=1}^{L_{s}}\sum_{r=1}^{N_{R}}{\left|H_{\mathrm{est}}\right|}^{2}}{\sum_{i=1}^{L_{s}}\sum_{r=1}^{N_{R}}{\left|H_{\mathrm{est}}-H_{\mathrm{mmse}}\right|}^{2}}\right), \end{array}$$

where $\hat{\gamma }$ represents the estimated SNR in dB, $P_{s}$ is the total power of the transmitted signal, and $L_{s}$ represents the number of symbols in the time domain.

## 4. Design of PE-MSRN

### 4.1. Network Architecture

**Figure 2:**
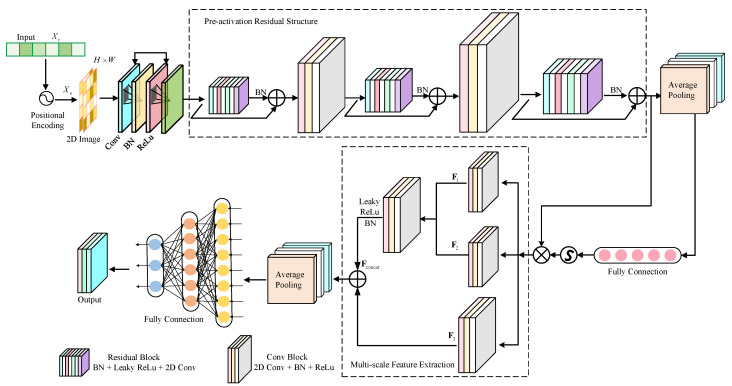
The proposed architecture of PE-MRSN algorithm.

The architecture of the proposed PE-MSRN algorithm is illustrated in Figure 2. By integrating residual connections and multi-scale feature extraction within a deep convolutional neural network, combined with position encoding-enhanced data preprocessing, the algorithm achieves robust deep learning of 5G signal features. The architecture comprises an image input layer, the convolution network, residual blocks, a squeeze-and-excitation (SE) attention network, a multi-scale feature extraction mechanism, and a fully connected output stage. Dynamic size adaptation and optimized training strategies significantly enhance positioning performance. The data processing pipeline within PE-MSRN can be summarized as follows: First, the raw 5G CSI features $X_{r}$ undergo an enhancement process, incorporating positional encoding for angular data and nonlinear transformations for time delay, resulting in an extended feature vector $X_{e}$. This vector, now rich with spatial information, is systematically reshaped into a 2D image-like format J with the size of H×W, making it amenable to convolutional processing. This image serves as the input to our deep network. The initial layers of the network perform primary feature extraction, followed by a series of residual blocks to learn deeper representations. Crucially, a multi-scale feature extraction module then processes the feature maps through parallel branches with different kernel sizes to capture both fine-grained details and broader contextual patterns simultaneously. Finally, these multi-scale features are fused and passed to fully connected layers to regress the final 3D coordinates. This hierarchical and multi-scale design is key to the high performance of the algorithm.

**Figure 3:**
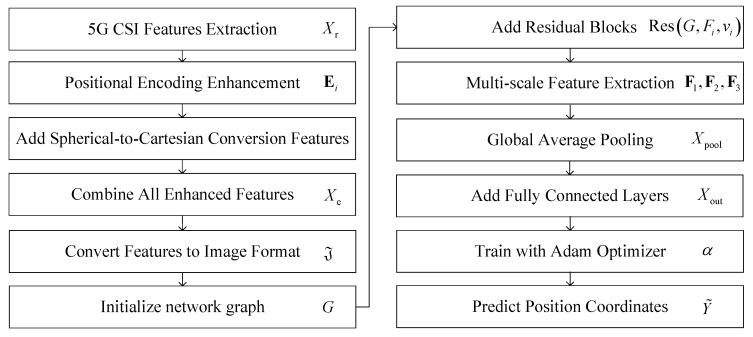
The flowchart of the PE-MRSN algorithm.

The algorithm flowchart of the proposed PE-MSRN algorithm is shown in Figure 3. The features $X_{e}$ are transformed into a single-channel feature map. This transformation enables the feature to be converted into a two-dimensional image input, making it easier to use convolution operations to capture spatial dependencies. These are first processed by an initial convolutional layer for primary feature extraction, followed by residual blocks employing convolutions with different sizes of channels, respectively, to extract spatial features while mitigating gradient vanishing.

The SE attention mechanism applies global average pooling, generating channel weights through fully connected layers with a compression ratio of β and sigmoid activation, thereby enhancing key feature responses and improving robustness to noise. The multi-scale feature extraction network captures diverse spatial patterns through parallel $F_{1}$, $F_{2}$, and $F_{3}$ convolutional branches, fuses them via a convolution, and optimizes gradient flow with residual connections. After dimensionality reduction through global average pooling, the features are processed by three fully connected layers with progressively reduced dimensions, including batch normalization, leaky rectified linear unit (LeakyReLU), and dropout, gradually reducing the dimensionality from high dimensions and finally outputting the normalized coordinates through a fully connected layer and a regression layer. The pseudocode of the proposed PE-MSRN algorithm is shown in Algorithm 1.
**Algorithm 1:** The PE-MSRN algorithm for 5G positioning.
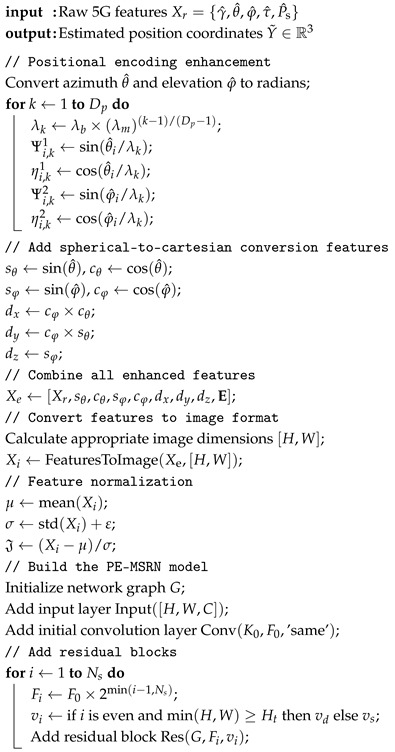

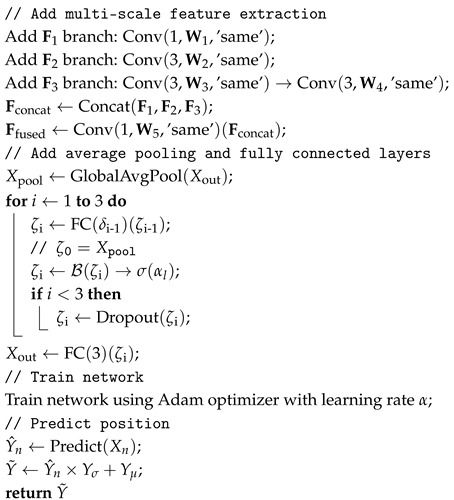


### 4.2. Design of Positional Encoding Algorithm

The basic idea of positional encoding combines the position-related encoding value with each input feature, typically generated using sine and cosine functions, thus providing a unique representation for each position. In this study, the positional encoding from the Transformer architecture is integrated and applied to the data preprocessing stage of 5G deep learning positioning [43]. By enhancing the 2D estimated angles $\hat{\theta }$ and $\hat{\phi }$, with positional encoding, the additional spatial information is provided to the network. This allows each pixel in the image to not only contain its own feature information but also its spatial position attributes. Additionally, by incorporating other cross-features related to AOA and TOA, the algorithm can achieve more accurate position estimation.

Firstly, the 2D estimated angles are converted to radians. The dimension after position encoding is divided into four parts, and the dimension of each part is defined as K, which are used for the sine and cosine encoding of the angles. For the k∈{1,2,…,K} frequency dimension, the corresponding $\lambda_{k}$ can be defined as

(5) $$\lambda_{k}=\lambda_{0}\times 10,000^{\frac{k-1}{K-1}},$$

where $\lambda_{0}$ is a reference initial value that determines the starting point of the frequency distribution and is used to control the frequency range of the position encoding. The definition of the frequency factor allows it to cover multiple scales, enabling the capture of positional features at different frequency ranges. Through a geometric progression, the frequency starts from a small value 2π and gradually increases until it reaches 10,000×2π, thereby spanning multiple scales in the frequency range [43,44] and capturing finer-grained angular variation information.

Secondly, for the *i*-th azimuth angle ${\hat{\theta }}_{i}$ and the elevation angle ${\hat{\varphi }}_{i}$, the corresponding sine and cosine terms for positional encoding can be illustrated as

(6) $$\left\{\begin{array}{c} \Psi_{i,k}^{1}=\sin\left(\frac{{\hat{\theta }}_{i}}{\lambda_{k}}\right),\Psi_{i,k}^{2}=\sin\left(\frac{{\hat{\varphi }}_{i}}{\lambda_{k}}\right)\\ \eta_{i,k}^{1}=\cos\left(\frac{{\hat{\theta }}_{i}}{\lambda_{k}}\right),\eta_{i,k}^{2}=\cos\left(\frac{{\hat{\varphi }}_{i}}{\lambda_{k}}\right) \end{array}\;k=1,\ldots ,K\right..$$

Finally, all the encodings for 2D estimated angles will be concatenated into position encoding vectors, as shown below

(7) $$E_{i}=\left[\Psi_{i,1}^{1},\ldots ,\Psi_{i,K}^{1},\eta_{i,1}^{1},\ldots ,\eta_{i,K}^{1},\Psi_{i,1}^{2},\ldots ,\Psi_{i,K}^{2},\eta_{i,1}^{2},\ldots ,\eta_{i,K}^{2}\right]\in R^{D}.$$

This part of the encoding captures the periodic variation of the angles. The frequency ranges at different scales allow the network to leverage the combination of low-frequency and high-frequency signals, enhancing its feature representation ability. To further utilize the geometric information of the angles, $\hat{\theta }$ and $\hat{\varphi }$ are used to compute the direction vector through trigonometric functions, as follows

(8) $$\left\{\begin{array}{c} s_{\theta_{i}}=\sin\left({\hat{\theta }}_{i}\right),c_{\theta_{i}}=\cos\left({\hat{\theta }}_{i}\right)\\ s_{\varphi_{i}}=\sin\left({\hat{\varphi }}_{i}\right),c_{\varphi_{i}}=\cos\left({\hat{\varphi }}_{i}\right) \end{array},\right.$$

and the spherical coordinates are converted to Cartesian coordinates to compute the unit direction vector, illustrated as

(9) $$d_{i}=\left[\begin{array}{c} d_{x}\\ d_{y}\\ d_{z} \end{array}\right]=\left[\begin{array}{c} c_{\varphi_{i}}\times c_{\theta_{i}}\\ c_{\varphi_{i}}\times s_{\theta_{i}}\\ s_{\varphi_{i}} \end{array}\right].$$

This directional quantity can directly represent the spatial direction of the signal transmission path, helping the network capture spatial features in different directions. The original feature package contains the information of the time delay $\hat{\tau }$; to further enrich the input features, a nonlinear transformation is applied to the time delay as follows

(10) $$\left\{\begin{array}{c} {\hat{\tau }}_{\log,i}=\log\left({\hat{\tau }}_{i}+\varepsilon \right)\\ {\hat{\tau }}_{sqrt,i}=\sqrt{{\hat{\tau }}_{i}+\varepsilon } \end{array},\right.$$

where ε represents a small adjustment factor to avoid some deviations. Finally, all features processed by positional encoding and transformation are integrated to form a new enhanced feature vector, which is then input into the network for training. The extended input dataset based on positional encoding features contains SNR estimation $\hat{\gamma }$, AOA values $\hat{\theta }$ and $\hat{\phi }$, time delay estimation $\hat{\tau }$, the received signal power ${\hat{P}}_{s}$, sine and cosine transformations of 2D angles, 3D unit direction vectors, time delay transformation, cross-characteristics of SNR and power, and positional encoding.

After the feature expansion operation described in the previous section, the input feature dimension becomes $X_{e}$. The image area is set to H×W≥N, where each feature value is mapped to a pixel in the image. The image area must be at least *N*. For a square image, the ideal image dimensions is calculated as follows

(11) $$H=W=\lceil \sqrt{N}\rceil ,$$

where $\left\lceil \sqrt{N}\right\rceil$ denotes the ceiling of the square root of *N*, ensuring the image width and height can accommodate all features. For each feature under each SNR value at every coordinate point, the feature index is denoted as $X_{e}\left[i\right],i\in \left[1,N\right]$, and its corresponding pixel coordinates in the image are (r,c), where *r* is the row index and *c* is the column index, calculated as follows

(12) $$\left\{\begin{array}{c} r=\left\lfloor \frac{i}{W}\right\rfloor \\ c=i-\left\lfloor \frac{i}{W}\right\rfloor \times W \end{array}\right.\;\;\;\;.$$

Given an image $J\in R^{H\times W\times C}$, where *H* and *W* represent the height and width and *C* is the number of channels, each feature $X_{e}\left[i\right]$ is assigned to the corresponding position in the image, as shown below

(13) $$J\left(r,c\right)=X_{e}\left[i\right].$$

The reference initial value $\lambda_{0}$ is a critical hyperparameter that was set to 2π in our experiments. This choice is based on the rationale that the fundamental frequency of encoding should correspond to a full cycle of the angular unit, allowing the subsequent geometric progression to capture variations from this base frequency up to very high frequencies, thus covering a comprehensive spectrum of positional information.

### 4.3. Residual Connection Mechanism

To enhance the training stability and representational capacity of deep neural networks, this study integrates a residual connection network based on the pre-activation structure into the PE-MSRN algorithm. This network consists of two convolutional layers, each followed by batch normalization (BN) and a LeakyReLU activation function. Compared with the conventional BN→ReLU→Conv arrangement, the pre-activation structure enables more effective gradient backpropagation in deep layers. The output of the residual block can be defined as

(14) $$y=F\left(x,\left\{W_{i}\right\}\right)+\Lambda \left(x\right).$$

When the input and output dimensions are consistent, an identity mapping is used; otherwise, a convolutional kernel is applied for dimensional alignment. The LeakyReLU activation function is used in the residual blocks to improve gradient flow and avoid neuron inactivation. Unlike standard ReLU, which outputs zero for all negative inputs, LeakyReLU allows a small negative slope α to preserve non-zero gradients, as given by

(15) $$\sigma \left(x\right)=\left\{\begin{array}{cc} x, & \mathrm{if}\;x\geq 0\\ \alpha x, & \mathrm{if}\;x<0 \end{array}\right..$$

This structure offers significant advantages during backpropagation. The gradient can bypass the nonlinear transformations through the identity shortcut, thereby alleviating vanishing gradient issues. Specifically, the derivative with respect to the input can be expressed as

(16) $$\frac{\partial L}{\partial x}=\frac{\partial L}{\partial y}\left(\frac{\partial F(x)}{\partial x}+I\right),$$

where L denotes the loss function and I is the identity matrix. This design not only improves the trainability of deep neural networks but also helps preserve the statistical distribution consistency between the input and the output features.

### 4.4. Multi-Scale Feature Extraction Mechanism

To more effectively capture the spatial diversity and scale variation inherent in the input features, a multi-scale feature extraction mechanism is designed in the proposed network. This network is embedded after the final residual block and extracts features across different receptive fields through parallel convolutional branches at multiple scales.

Specifically, this block consists of three parallel branches: the first branch employs a $F_{1}$ convolution to extract local fine-grained features; the second branch applies a $F_{2}$ convolution to extract mid-scale spatial representations; and the third branch employs a larger $F_{3}$ convolution, enabling the capture of more global spatial patterns. Each convolutional path is followed by a BN operation and a LeakyReLU activation function, which help accelerate convergence and enhance nonlinear representational capacity. The multi-scale feature extraction process of the three branches can be expressed as follows

(17) $$\left\{\begin{array}{c} F_{1}=\sigma \left(B_{1}\left(W_{1}*X\right)\right)\\ F_{2}=\sigma \left(B_{2}\left(W_{2}*X\right)\right)\\ F_{3}=\sigma \left(B_{4}\left(W_{4}*\sigma \left(B_{3}\left(W_{3}\times X\right)\right)\right)\right) \end{array}\right.,$$

where $X\in R^{H\times W\times C}$ denote the input feature map of the network, ∗ represents the convolution operation, and $W_{i}$ denotes the convolution kernel of the *i*-th layer. $B_{i}\left(\cdot \right)$ is the batch normalization operation, and σ(·) denotes the LeakyReLU activation function. The outputs from the three parallel branches are concatenated along the channel dimension to form a unified feature representation, as given by

(18) $$F_{\mathrm{concat}}=\left[F_{1},F_{2},F_{3}\right]\in R^{H\times W\times 3C}.$$

To integrate multi-scale information and reduce dimensionality, the concatenated features are fused using a 1×1 convolution, as followed by

(19) $$F_{\mathrm{fused}}=\sigma \left(B_{5}\left(W_{5}*F_{\mathrm{concat}}\right)\right).$$

To preserve the original information and enhance gradient flow, a shortcut connection is introduced, and the final output of the network is then obtained by

(20) $$Y=\sigma \left(F_{\mathrm{fused}}+B_{s}\left(W_{s}*X\right))\right),$$

where $W_{s}$ is a 1×1 convolution kernel. The proposed multi-scale feature extraction mechanism enables parallel modeling with different receptive fields, allowing joint learning of features at multiple spatial resolutions. On the one hand, small-scale convolutions help capture fine-grained geometric structures, such as localized directional variations; on the other hand, large-scale paths are capable of capturing spatial correlations across regions, thereby enhancing the network’s ability to accurately estimate position information in complex environments.

### 4.5. Introduction of Comparison Algorithms

To demonstrate the effectiveness of the proposed innovative algorithm, we introduce four other algorithms currently used in deep learning. Algo.3, Algo.4, and Algo.5 are based on feedforward neural networks composed of fully connected layers, while Algo.1 (PE-MSRN) and Algo.2 are built on CNN with different input feature approaches. Through a systematic comparison of the performance of these five different deep learning architectures in 5G signal indoor positioning tasks, we prove the effectiveness of the proposed positional encoding method and network. The specific network architectures and the parameters of networks are shown in Table 2, and the detailed architectures of the five introduced algorithms are as follows

- PE-MSRN (Algo.1): As shown in Figure 2, this algorithm contains various sizes of residual blocks, a global pooling layer, and fully connected layers. Each residual block contains two convolutional layers and a skip connection. It integrates SE channel attention networks to dynamically adjust feature channel weights and employs a multi-scale feature extraction strategy by processing features in parallel through $F_{1}$, $F_{2}$, and simulated $F_{3}$ convolutions. Different scales of information are integrated through a feature fusion layer, forming a network structure that is both deep and wide.
- CNN (Algo.2): The classic CNN architecture for processing 5G signal features converted into image format is proposed, which includes multiple convolutional blocks, pooling layers, and fully connected layers. Each convolutional block includes convolution, batch normalization, and activation functions. In contrast to Algo.1, which lacks innovations like positional encoding, residual connections, and multi-scale feature extraction, the traditional CNN architecture is used to evaluate its performance for 5G deep learning localization.
- FCNet (Algo.3): The fully connected network (FCNet) employs a simple yet effective feedforward neural network structure, consisting of residual blocks based on self-attention mechanisms and fully connected layers. On the basis of Algo.1, the convolutional network is simplified into a fully connected network, without positional encoding and multi-scale feature extraction. It includes multiple residual blocks based on the attention mechanism, capturing the relationships between signal features through self-attention. This algorithm can assess the effectiveness of the attention mechanism and residual connections in 5G localization.
- FE-FCNet (Algo.4): The input feature-enhanced fully connected network (FE-FCNet) removes positional encoding, convolutional structure, and multi-scale feature extraction mechanisms compared with Algo.1. It introduces LeakyReLU activation functions and creates richer input features through feature engineering, such as trigonometric transformations and composite features of power and delay. And it improves upon Algo.3 by increasing network depth and expanding hidden layer dimensions. This allows for further evaluation of the contribution of feature expansion processing of input data to the performance of the fully connected network.
- PE-FCNet (Algo.5): The positional encoding-enhanced fully connected network (PE-FCNet) maintains the same overall architecture as Algo.4, and it innovatively introduces positional encoding for feature expansion and attention mechanisms with tanh activation. It employs a higher dropout rate to enhance regularization. It removes the convolutional layers, attention mechanism, and residual structure of Algo.1, evaluating the contribution of positional encoding to spatial information representation in a simplified architecture.

### 4.6. Complexity Discussion of Different Algorithms

For the deep neural network, the computation complexity mainly depends on three key components: input layer, hidden layer (including attention mechanism and residual blocks), and output layer. The following summarizes the computation complexity of each part and outputs the total computation cost. The variable notations and corresponding explanations can be referenced to Table 1 as above.

For each single training epoch, the primary computation of the input layer involves both the convolution and the fully connected layers. The calculation of the input layer mainly includes two parts: matrix multiplication and bias addition. For each batch of data, the full connection calculation complexity of the input layer can be expressed as

(21) $$C_{i}=2\times \Omega \times D\times \delta .$$

The residual integration involves self-attention mechanisms and residual connections, with multiple iterations of operations and additive operations for residual connections. For single-layer attention residuals, the main channels involve two types of fully connected layers and attention-based structures, and the total computation can be calculated as

(22) $$C_{b}=3\times \Omega \times \delta^{2}+2\times \Omega \times \delta .$$

The output layer is responsible for mapping the high-dimensional feature representations to the final prediction results, and its computation complexity is directly related to the hidden dimension and output dimension, as given by

(23) $$C_{o}=2\times \Omega \times \delta \times \rho .$$

Thus, the total computation complexity for a single training epoch can be computed as

(24) $$\begin{array}{cc} C_{\mathrm{epoch}\;} & =\frac{N}{\Omega }\times \left(C_{i}+\xi \times C_{b}+C_{o}\right)\\ \; & =\frac{N}{\Omega }\times \left(2\Omega D\delta +\xi \left(3\Omega \delta^{2}+2\Omega \delta \right)+2\Omega \delta \rho \right)\\ \; & =N\times \left(2D\delta +3\xi \delta^{2}+2\xi \delta +2\delta \rho \right). \end{array}$$

For Algo.5 (PE-FCNet), with D=32, δ=512, ξ=4, and ϵ=100, the specific calculation of the computational complexity is $3.19\times 10^{8}\times N$. And for convolutional networks, its computational complexity is related to the size of the convolution kernel, the size of the input feature map, the size of the output feature map, and the number of convolution kernels. For a single training round, the computational complexity of the convolution layer in the network can be expressed as

(25) $$C_{\mathrm{conv}\;}^{i}=2\times Q_{i}\times R_{i}\times \varpi_{i}\times W_{i}^{2}\times \gamma_{i}\times \Omega .$$

Each convolution kernel performs a convolution operation with the input feature map and calculates the weighted sum of multiple local regions. Therefore, the complexity of the convolution layer is directly related to the size of the feature map and the number of convolution kernels. The fully connected layer is used for feature integration. Its computational complexity is mainly related to the dimension of the input feature and the number of output nodes. The computational complexity of the j-th layer of the fully connected layer can be calculated as

(26) $$C_{\mathrm{fc}}^{j}=2\times D_{j}\times \rho_{j}\times \Omega .$$

The total training computational complexity can be defined as

(27) $$C_{\mathrm{total}}=\epsilon \times \frac{N}{\Omega }\times \left(\sum_{i=1}^{L_{\mathrm{conv}}}2Q_{i}R_{i}\varpi_{i}W_{i}^{2}\gamma_{i}+\sum_{j=1}^{L_{fc}}2D_{j}\rho_{j}\right).$$

Algo.1 (PE-MSRN) consists of approximately $L_{\mathrm{conv}}=5$ convolutional layers and $L_{\mathrm{fc}}=3$ fully connected layers. The network reduces the image size $Q_{i}^{1},R_{i}^{1}$ gradually from 64×64 to 8×8 and increases the kernel size $W_{i}^{1}=\left\{3\times 3,5\times 5\right\}$ to extract richer features. The output channels $\gamma_{i}^{1}$ range from 64 to 512, with the final output $\rho_{j}^{1}=3$. $\varpi_{i}^{1}$ increases layer by layer from 1 to 512 at the deepest layer, and $D_{j}^{1}=8\times 8\times 512$. In contrast, Algo.2 (CNN) has fewer convolutional layers, with $L_{\mathrm{conv}}=3$ and $L_{\mathrm{fc}}=3$. The kernel size $W_{i}^{2}$ is fixed as 3×3, and the output channels $\gamma_{i}^{2}$ range from 32 to 256. $D_{j}^{2}$ is set to 8×8×256. The input channel number $\varpi_{i}^{2}$ progressively increase from a single channel to 256 through the network depth.

For Algo.3 (FCNet), the specific parameter configuration is ϵ=200, the input feature dimension $D^{3}=5$, the number of neurons in the hidden layer $\delta^{3}=64$, the number of residual attention blocks $\xi^{3}=2$, and the output dimension $\rho^{3}=3$. Algo.4 (FE-FCNet) is configured as ϵ=80, $D^{4}=19$, $\delta^{4}=512$, $\xi^{4}=4$, and $\rho^{4}=3$. Algo.5 (PE-FCNet) uses ϵ=100, $D^{5}=32$, $\delta^{5}=512$, $\xi^{5}=4$, and $\rho^{5}=3$. Substituting the network parameters into the complexity calculation formulas of the above different algorithms, the numerical analysis of the network training operation amount can be obtained as shown in Table 3.

## 5. Simulation Results and Discussion

### 5.1. Simulation Setting

To comprehensively evaluate the performance of the proposed algorithm in 5G positioning under different scenarios, we design a rigorous simulation environment. The simulation parameters are shown in Table 4, $N_{\mathrm{SRS}}^{\mathrm{symbol}}$ represents the number of time domain symbols of SRS signal, $N_{\mathrm{SRS}}^{\mathrm{PRB}}$ indicates the size of frequency domain resources, and $N_{\mathrm{SRS}}^{\mathrm{comb}}$ represents the comb size of frequency–domain. All experiments are based on 5G SRS signals generated according to the 3GPP 38.211 standard [45], using the FR2 millimeter-wave frequency band at 30 GHz and subcarrier spacing of 60 kHz, with the SRS signal configured as two symbols in the time domain and 262 RBs in the frequency domain. To save bandwidth, the comb size of the SRS is configured to four, and the number of receiving antennas is set to 64. The simulations are conducted on a workstation equipped with an NVIDIA GeForce GTX 1650, Core™ i5-9300H CPU, and 512 GB of RAM using MATLAB R2022b.

Compared with Algo.3, the complexities of Algo.4 and Algo.5 increase by approximately 49 times and 1.26 times, respectively. For Algo.1 and Algo.2, the training complexity mainly comes from convolutional layers and fully connected layers. It is estimated that the computational complexities of Algo.1 and Algo.2 are approximately $9.40\times 10^{8}\times N$ and $2.00\times 10^{8}\times N$, respectively. The complexity of Algo.1 is about 4.7 times that of Algo.2.

The training dataset is constructed from a database collected in a 5G link-level simulation environment, containing five basic features: SNR, AOA, TOA, and RSSI, as well as corresponding precise 3D coordinate labels. The dataset is divided into 70% training set, 15% validation set, and 15% test set. To evaluate the algorithm performance under different SNR conditions, noise samples ranging from −10 dB to 6 dB are added to the test set. All the networks use the Adam optimizer, the initial learning rate is set to 0.001, and the learning rate decay strategy is used. To prevent overfitting, regularization techniques such as batch normalization and dropout (0.2–0.3) are applied, and the L2 regularization coefficient is $1\times 10^{-4}$ to $1\times 10^{-5}$.

After cross-validation, Algo.3 (FCNet) is trained for 200 epochs and Algo.4 (FE-FCNet) for 80 epochs, while Algo.1 (PE-MSRN), Algo.2 (CNN), and Algo.5 (PE-FCNet) are trained for 100 epochs. The convergence of training is validated through loss and validation curves, and the effectiveness of the designed networks is verified by ablation experiments. The root mean square error (RMSE), average error, and other indicators were used to measure the positioning accuracy of the network under different noise conditions in different scenarios. The three specific scenario requirements verified in the simulation are shown in Table 5.

### 5.2. Discussion of Convergence Performance

**Figure 4:**
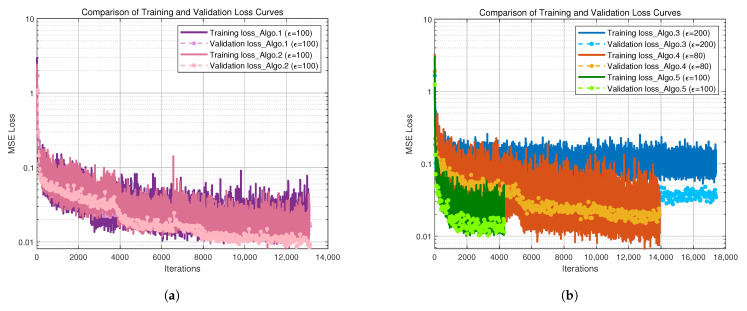
Convergence performance curve comparison of different algorithms. (**a**) Convergence performance curves of Algo.1 and Algo.2. (**b**) Convergence performance curves of Algo.3, Algo.4, and Algo.5.

For the 3D indoor autonomous driving localization scenario, further simulation analysis of the algorithm convergence performance was conducted using a 5G measurement dataset with a partition step size of two. Figure 4 illustrate the trends of training loss versus iteration number for each algorithm during training. The mean squared error (MSE) was chosen as the loss function because it can intuitively reflect the overall deviation between predicted and true values and effectively evaluate the convergence of the network. Compared with Algo.3 and Algo.4, the fully connected network based on positional encoding (Algo.5) achieves lower training loss with fewer iterations, demonstrating faster convergence speed and higher training efficiency, as shown in Figure 4b. In the comparison between Algo.1 and Algo.2, the final training and validation losses of Algo.2 are 0.0321 and 0.0163, respectively, whereas Algo.1 achieves even smaller training and validation losses of 0.0145 and 0.0091, as illustrated in Figure 4a. This improvement is attributed to a more effective feature fusion strategy and optimization scheduling of the PE-MSRN algorithm, including a more reasonable learning rate decay and adapted activation function selection, which help enhance gradient flow efficiency and training stability. These factors further indicate that Algo.1 attains better fitting and generalization during training.

**Figure 5:**
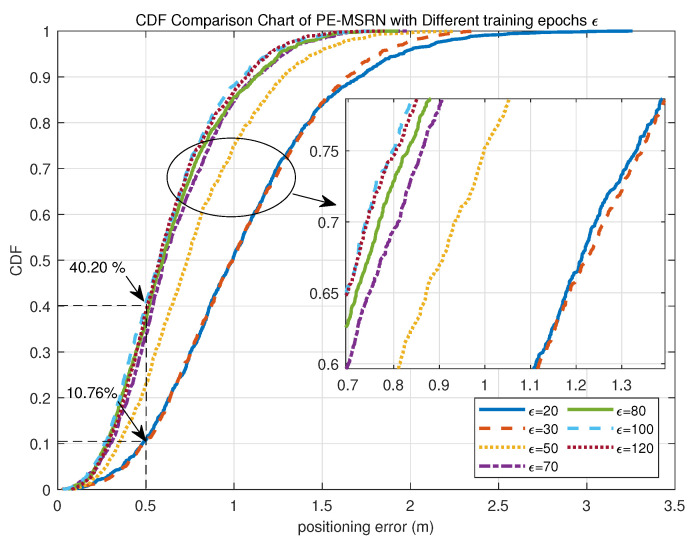
CDF comparison curves of the PE-MSRN algorithm with different training epochs ϵ.

As shown in Figure 5, the cumulative distribution function (CDF) of the proposed PE-MSRN algorithm improves with increasing training epochs, reaching peak performance at 100 epochs, where the validation set accuracy achieves a CDF(80%) = 0.78 m. Through comprehensive analysis of both the CDF curve and the training loss curve, it is evident that the PE-MSRN algorithm can achieve lower error levels and stable convergence to smaller losses compared with shallow fully connected networks and basic CNN, thereby improving the training efficiency and localization accuracy of 5G deep learning-based positioning.

To quantitatively evaluate the convergence behavior of the proposed algorithm under different training epochs, a comparative analysis of its positioning performance is conducted on the test set at 20, 30, 50, 70, 80, 100, and 120 epochs, as summarized in Table 6. The primary evaluation metrics include mean positioning error, 90% percentile error, maximum error, the proportion of sub-meter, and the proportion of sub-half-meter estimates.

As the number of training epochs increases, the positioning accuracy of the algorithm improves consistently. For instance, the mean positioning error decreases from 1.0476 m at 20 epochs to 0.6178 m at 100 epochs, representing a 40.99% reduction. Similarly, the 90th percentile error drops from 1.9507 m to 1.2137 m, reflecting an improvement of approximately 37.78%. The maximum error also shows a significant decline, from 3.2585 m to 1.7694 m, amounting to a 45.69% reduction.

In terms of positioning accuracy distribution, the proportion of sub-meter predictions increases substantially from 50.96% at 20 epochs to 88.07% at 100 epochs, an improvement of 37.11%. Likewise, the proportion of predictions within 0.5 m rises from 10.76% to 40.20%, indicating an enhancement of 29.44%. These results demonstrate that with more training, the algorithm becomes increasingly capable of delivering high-precision location estimates with greater stability. When extended to 120 epochs, the mean error slightly increases to 0.6307 m, suggesting a potential onset of overfitting. Therefore, under the current experimental setting, 100 training epochs represent the optimal configuration for the PE-MSRN algorithm, balancing low positioning error with the highest proportion of sub-meter accuracy.

### 5.3. Discussion of Ablation Performance

In order to further verify the innovations proposed in this paper: feature expansion based on position encoding, a residual connection network and multi-scale feature extraction mechanism, and the contribution of each network to the algorithm positioning performance, a systematic ablation experiment was designed for performance comparison. All networks were trained under the same training configuration: using the Adam optimizer, the initial learning rate was 0.001, the fixed training epochs are fixed at 100, the round batch size was 64, and the mean square error (MSE) was used as the loss function. The comparison algorithms is defined as follows:

- PE-MSRN: The multi-scale residual network based on position encoding is the complete algorithm proposed in this paper.
- PE-MSN: The PE-MSRN algorithm without a residual connection network.
- PE-SRN: The PE-MSRN algorithm without multi-scale feature extraction.
- MSRN: The PE-MSRN algorithm without position encoding for feature expansion preprocessing.

**Figure 6:**
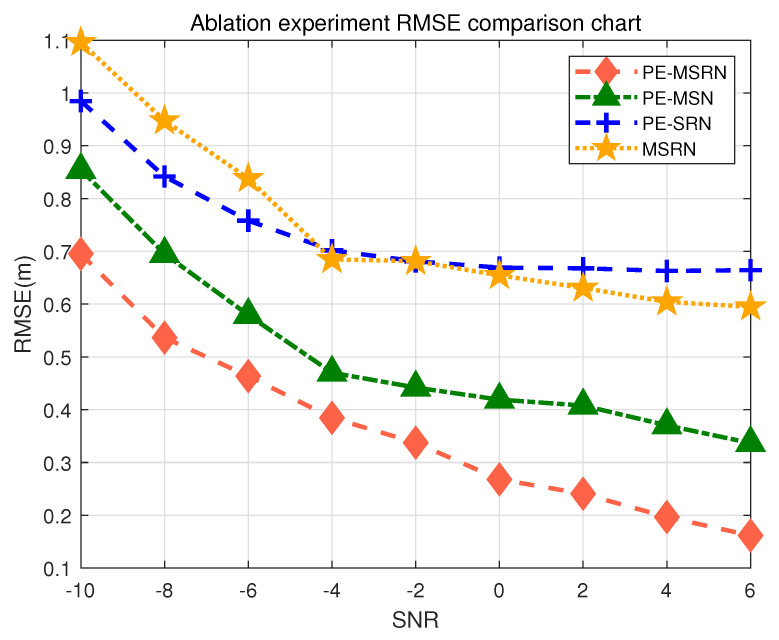
Comparison of positioning accuracy of different algorithms in the ablation experiment.

The 3D positioning accuracy performance of different algorithms under various SNR conditions is illustrated in Figure 6. The trained networks were simulated in a real measurement environment, with RMSE values being statistically computed under 1000 Monte Carlo simulations at different SNR levels. As the SNR increases, the errors of all algorithms decrease, reflecting that improved signal quality contributes to enhanced positioning accuracy, with stabilization occurring after the SNR of 2 dB. The performance of different algorithms on the validation set is shown in Table 7, which compares positioning accuracy across various metrics, including mean error, maximum error, and others. The proposed PE-MSRN algorithm achieves the highest positioning accuracy, reaching 40 cm in the SNR of −4 dB, and approximately 15 cm in the SNR of 6 dB. In Table 7, the proposed algorithm achieves a mean positioning accuracy of 0.6178 m on a 15% validation set, with sub-meter-level accuracy at 72%, meeting the high-precision localization requirements for autonomous robots in indoor 3D spaces. This demonstrates that the integration of residual structures, multi-scale convolutions, and positional encoding in the complex feature extraction and fusion strategy greatly enhances the positioning accuracy.

The PE-MSN algorithm, with the removal of the residual connection block, shows a 52% decrease in RMSE compared with the complete algorithm, with a practical measurement error of 0.32 m, confirming the critical role of residual blocks in alleviating gradient degradation during training and enhancing the performance of deep networks. The residual connection network enables the network to access features from earlier layers even in deep layers, strengthening the ability of the algorithm to express multi-scale and multi-level features. In 5G localization, spatial information (such as AOA) and temporal information (such as TOA) need multi-level fusion. The residual connection ensures that these features are not lost as the network deepens. The PE-SRN algorithm, with the removal of multi-scale feature extraction, shows a 75% decrease in accuracy, with RMSE approaching 0.6 m as SNR increases, resulting in overall higher errors. The multi-scale branches, compared with a single convolution kernel, allow the algorithm to learn both local and global features by combining convolutions with different receptive fields.

Particularly noteworthy is the performance of the MSRN model, showing a 77% decrease in accuracy, which lacks the positional encoding module. This model exhibits the most significant performance degradation, with its mean error increasing by approximately 30% compared with the full PE-MSRN model and its sub-meter accuracy dropping by over 16%. This result provides strong empirical evidence for our central hypothesis: that explicitly injecting spatial priors via positional encoding is fundamental to achieving high accuracy. Without this encoding, the network treats CSI features as an unordered collection of values, failing to leverage the inherent geometric relationships contained within the AOA and TOA data. The significant performance drop confirms that the convolutional layers alone, while powerful, are insufficient to learn these spatial relationships from scratch, underscoring the critical contribution of our feature engineering approach.

In summary, the ablation study results thoroughly demonstrate the positive contribution of each key network to the algorithm’s performance improvement, validating the effectiveness of the proposed network architecture.

### 5.4. Performance Discussion in 2D Scenarios

**Figure 7:**
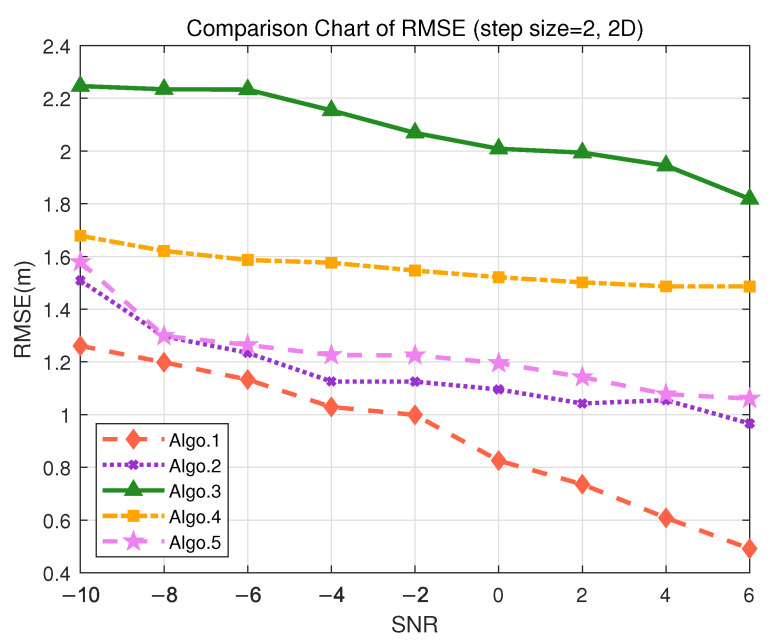
Comparison of RMSE performance in the S3 (2D) scenario. (Algo.1: PE-MSRN; Algo.2: CNN; Algo.3: FCNet; Algo.4: FE-FCNet; Algo.5: PE-FCNet.).

To evaluate the positioning performance of different algorithms in 2D localization scenarios, we conducted simulation experiments under two representative use cases: indoor trajectory tracking (requiring accuracy < 50 cm) and tool tracking (requiring accuracy < 2 m). In autonomous driving, it is essential to measure the 2D trajectory indoors, where high accuracy is crucial for positioning. Given the different tolerance levels for localization error in these scenarios, a 2D grid environment is designed using granularity of 2 m and 10 m, respectively. The use of root mean square error (RMSE) to calculate positioning errors can comprehensively reflect the average error level across multiple simulations, providing an intuitive representation of positioning accuracy. As shown in Figure 7, the performance of various algorithms under different SNR conditions is illustrated for the granularity of 2 m.

The proposed PE-MSRN algorithm maintains sub-meter accuracy even under low SNR conditions, achieving approximately 40 cm accuracy when SNR = 6 dB. The RMSE consistently decreases with increasing SNR, indicating the robustness and stability of the algorithm in high-density trajectory scenarios. This makes it suitable for continuous path tracking of personnel or mobile robots. Notably, Algo.1 (PE-MSRN) and Algo.2 (CNN), both based on the deep convolutional networks, outperform other algorithms built on fully connected networks or attention mechanisms. The quantitative results for each algorithm in the 2D scenario are summarized in Table 8. The PE-MSRN algorithm achieves a mean localization error of 0.5703 m, reflecting an approximate 11% improvement over the baseline CNN algorithm.

**Figure 8:**
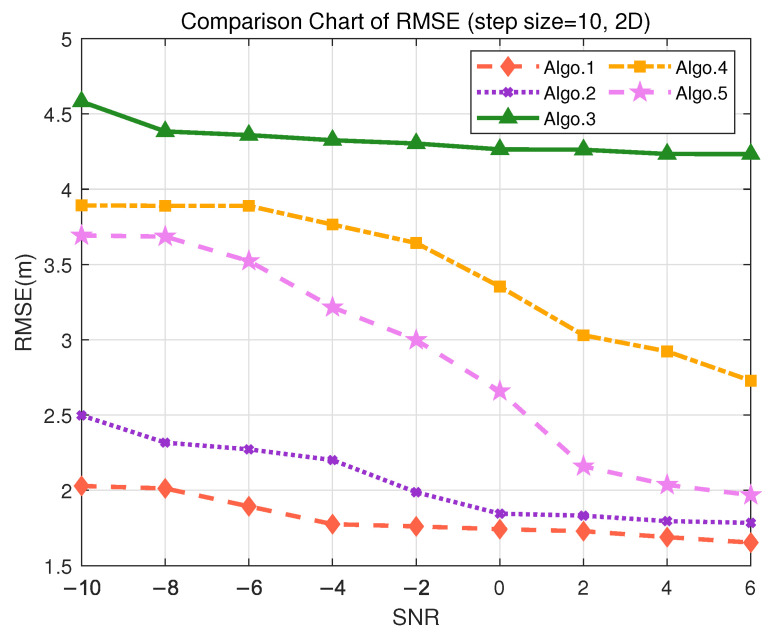
Comparison of RMSE performance in the S2 (2D) scenario. (Algo.1: PE-MSRN; Algo.2: CNN; Algo.3: FCNet; Algo.4: FE-FCNet; Algo.5: PE-FCNet.).

In the tool tracking scenario with a granularity of 10 m, the coarser grid leads to increased localization errors across all algorithms due to the sparser training data. As shown in Figure 8, Algo.1 still maintains a relatively low mean error of 1.6498 m, with a minimum error of approximately 5 cm, demonstrating its robustness and noise resilience under sparse sampling, as detailed in Table 9. The PE-MSRN algorithm meets the 2D localization accuracy requirements of smart factory environments. Algo.5 (PE-FCNet) also exhibits stable performance in this setting, consistently achieving 2 m accuracy at higher SNR levels. In contrast, Algo.3 (FCNet) and Algo.4 (FC-FCNet) have mean localization errors exceeding 3 m, while Algo.5 outperforms Algo.4 by around 30%, suggesting that the introduction of the positional encoding significantly enhances the feature learning capability of the network. Furthermore, Algo.2 improves upon Algo.5 by approximately 11.8%, indicating that convolutional networks possess superior spatial feature extraction capabilities, which effectively enhance localization accuracy.

### 5.5. Performance Discussion in 3D Scenarios

**Figure 9:**
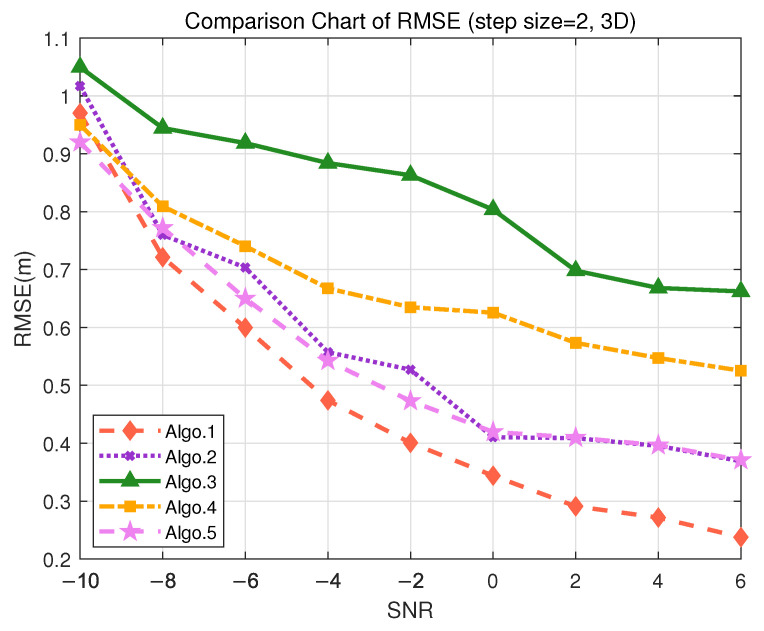
Comparison of RMSE performance in the S3 (3D) scenario. (Algo.1: PE-MSRN; Algo.2: CNN; Algo.3: FCNet; Algo.4: FE-FCNet; Algo.5: PE-FCNet.).

**Figure 10:**
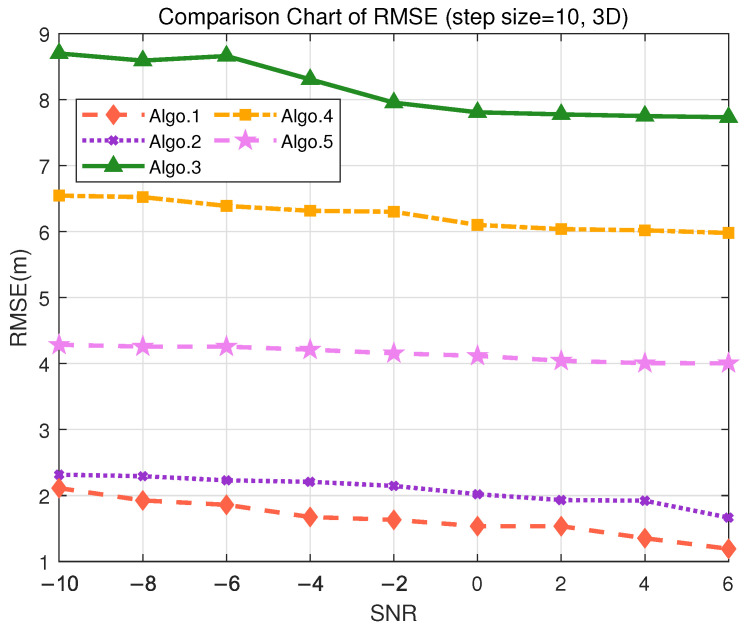
Comparison of RMSE performance in the S2 (3D) scenario. (Algo.1: PE-MSRN; Algo.2: CNN; Algo.3: FCNet; Algo.4: FE-FCNet; Algo.5: PE-FCNet.)

**Figure 11:**
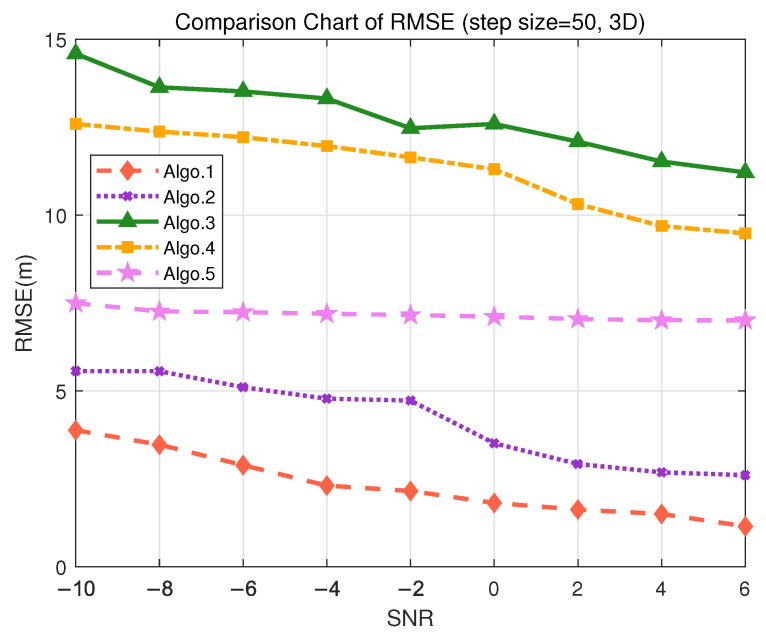
Comparison of RMSE performance in the S1 (3D) scenario. (Algo.1: PE-MSRN; Algo.2: CNN; Algo.3: FCNet; Algo.4: FE-FCNet; Algo.5: PE-FCNet.).

In order to demonstrate that the proposed algorithm can meet the positioning accuracy requirements for different scenarios, simulation experiments were conducted using the trained deep learning algorithm. Detailed simulation analysis was carried out for three 3D positioning scenarios: indoor autonomous driving and asset management, tool tracking in smart factories, and highway traffic flow management, under datasets with different granularity. The positioning performance analysis for each scenario is as follows:

- In the scenario of indoor autonomous driving and asset management positioning, the positioning accuracy is typically required to be within 30 cm to achieve precise navigation and logistics tracking, especially for the precise requirements of 3D positioning. For logistics tracking in factories, precise 3D positioning is necessary to accurately track the locations of different goods. During the data collection, the granularity of 2 m was used to divide the grid for offline training. The positioning performance of different algorithms is represented by RMSE, as shown in Figure 9, where RMSE decreases as SNR increases. Figure 9 indicates that in 3D positioning, at an SNR of 6 dB, the accuracy can reach 20 cm. Validation on the test set demonstrates that the PE-MSRN algorithm exhibits the smallest mean error of 0.5728 m in indoor 3D scenarios, as shown in Table 10, with a sub-meter-level accuracy rate of up to 90%, outperforming both the fully connected network and the basic CNN.
- Considering the smart factory tool tracking scenario, the positioning requirements are relatively high, with an accuracy demand of less than 4 m and a moderate tolerance for error. During the offline training phase, the granularity of 10 m was used to divide the grid for data collection. As shown in Figure 10, both Algo.2 and the proposed Algo.1 achieve positioning accuracy below 3 m in the real measurment. The PE-MSRN algorithm continues to exhibit excellent performance in this scenario, with a mean error of 1.2915 m, a 90% error of 2.2526 m, and a maximum error of 4.0681 m, as shown in Table 11. Compared with Algo.5, which is based on positional encoding, PE-MSRN improves by 40.83%, and it outperforms the Algo.2 by approximately 9%. Due to the larger grid size, all algorithms experience a degree of error increase. However, even under low SNR conditions, the PE-MSRN algorithm still demonstrates relatively small positioning errors, ranging from 1 m to 2.2 m.
- For highway traffic flow management, the accuracy requirements are relatively relaxed, but effective traffic management still needs to be ensured, with accuracy required to be within 10 m. The granularity of 50 m was used to divide the grid. As shown in Figure 11, the PE-MSRN algorithm achieves a positioning accuracy of approximately 2 m, and Algo.2 achieves an accuracy of around 3 m, in environments with the SNR being greater than 2 dB, while the positioning error of the other algorithms exceeds 7 m. The PE-MSRN algorithm still demonstrates superior performance in this scenario, with positioning metrics being shown in Table 12, where the mean error is 5.8188 m, the 90% error is 9.2305 m, and the maximum error is 20.2470 m. Despite the larger error at this step size, PE-MSRN maintains relatively small errors and shows more stable performance compared with other algorithms. For instance, Algo.3 has a mean error of 12.6442 m and a maximum error of 33.4930 m, which is significantly higher than PE-MSRN. Due to the larger grid granularity, the error growth in other algorithms is also more pronounced, whereas PE-MSRN demonstrates greater robustness, especially under low SNR conditions. The variation in errors is smaller, and the positioning accuracy remains within 5 m, meeting the requirements of the scenario.

In conclusion, whether it be applied for planar and three-dimensional positioning in indoor autonomous driving and asset management, tool tracking in smart factories, or highway traffic flow management, the PE-MSRN algorithm is capable of meeting the accuracy requirements for each scenario. This algorithm consistently provides stable and accurate positioning results under various step sizes and SNR conditions.

## 6. Conclusions

This paper has introduced and validated PE-MSRN, an innovative deep learning framework for high-precision 5G positioning in challenging GNSS-denied environments. By integrating a positional encoding scheme with a multi-scale residual network, our model effectively addresses the limitations of existing methods in capturing and processing complex spatial information from CSI data. The core innovation of this algorithm lies in the effective use of residual connections and multi-scale feature extraction strategies, which enhance the deep learning capabilities of the network and improve its performance when dealing with complex data. Experimental results show that PE-MSRN demonstrates high positioning accuracy in multiple real-world scenarios and exhibits excellent stability and robustness under different SNR and grid precision conditions. In scenarios with fine-grained grid partitioning, the proposed network can achieve sub-meter positioning accuracy even under low SNR conditions and reach positioning accuracy below 30 cm in ideal environments with higher SNR.

Overall, the PE-MSRN algorithm provides a novel solution to high-precision positioning problems, showing great potential in various application scenarios of remote sensing such as indoor autonomous driving, asset management, smart factories, and highway traffic flow management. Although PE-MSRN shows good performance in various scenarios, there is still room for improvement in high-dynamic or complex environments. In actual deployment, to reduce the power consumption of online measurements, this method relies on offline data collection and training. Although this approach helps reduce energy consumption during real-time operations, it may limit the system’s ability to quickly adapt to dynamic or changing environments.

## Figures and Tables

**Table 1:** Notation and operation descriptions.

Notation	Explanation
*H*	The length of the input image
*W*	The width of the input image
$X_{r}$	The CSI measurements
$X_{e}$	The extended features after position encoding
$\hat{\theta }$	The estimation of azimuth angle by SRS
$\hat{\phi }$	The estimation of elevation angle by SRS
$H_{\mathrm{est}}$	Channel response based on the least squares estimation
$H_{\mathrm{mmse}}$	Channel response based on the minimum mean square error estimation
*S*	The original transmitted signal
$f_{c}$	The center frequency of the orthogonal frequency division multiplexing (OFDM) signal
*V*	Noise
μ	The subcarrier spacing
*M*	The inverse fast fourier transform (IFFT) number size
${\hat{P}}_{s}$	The estimation of the received signal power
$P_{n}$	The noise power
$\hat{\gamma }$	The estimation of the SNR by SRS
λ	Frequency scaling factor in position encoding
K	Dimension number of the various parts of the positional encoding
J	The 2D image obtained by the extended features
*N*	Total number of training samples
F(·)	The nonlinear transformation operation in residual connections
Λ(·)	The shortcut paths in residual connections
σ(·)	The LeakyRelu activation function
B	The batch normalization operation
ϵ	Number of training epochs
Ω	Batch size in network training
*D*	Dimension number of the input feature for fully connected networks
ρ	Dimension number of the output feature for fully connected networks
δ	Neurons number in hidden layers of fully connected networks
$N_{s}$	The upper limit parameter controlling filter growth in ResNet block
$v_{s}$	The standard step size of the residual block
$v_{d}$	The downsampling step size of the residual block
$H_{t}$	The height threshold used to decide whether to downsample
*F*	The number of convolution kernels in the residual block
ζ	The output of the fully connected layer in CNN
ξ	Number of residual attention blocks in fully connected networks
$L_{\mathrm{conv}}$	Number of convolutional layers in CNN
$L_{\mathrm{fc}}$	Number of fully connected layers in CNN
R	The width of the output features in different convolutional layers
Q	The height of the output features in different convolutional layers
ϖ	Number of input channels in CNN
γ	Number of output channels in CNN
Γ	The spatial spectrum in multiple signal classification (MUSIC)
w	The matrix of array response
U	The matrix of eigenvectors of the noise covariance vector
W	Kernel size in CNN
F	The multi-scale feature extraction branch

**Table 2:** Comparison of network structures in different algorithms for 5G positioning.

Algorithm	Input	Activation Function	Hidden Dimensions	Network Depth	FC Layers
Algo.1	J	LeakyReLU	{512,256,128}	$L_{\mathrm{conv}}=5$, ϖ={64,128,256}	$L_{\mathrm{fc}}=3$
Algo.2	J	LeakyReLU	512	$L_{\mathrm{conv}}=3$, ϖ={32,64,128}	$L_{\mathrm{fc}}=3$
Algo.3	$X_{r}$	ReLU/LeakyReLU	64	ξ=2	$L_{\mathrm{fc}}=3$
Algo.4	$X_{e}$	ReLU	{512,256}	ξ=4	$L_{\mathrm{fc}}=4$
Algo.5	$X_{e}$	LeakyReLU	{512,256,128}	ξ=4	$L_{\mathrm{fc}}=6$

Algo.1: PE-MSRN; Algo.2: CNN; Algo.3: FCNet; Algo.4: FE-FCNet; Algo.5: PE-FCNet.

**Table 3:** Complexity comparison of different algorithms.

Algorithm	General Expression	Specific Complexity
Algo.1	$\epsilon \times \frac{N}{\Omega }\times \left(\sum_{i=1}^{L_{\mathrm{conv}}}2Q_{i}^{1}R_{i}^{1}\varpi_{i}^{1}{\left(W_{i}^{1}\right)}^{2}\gamma_{i}^{1}+\sum_{j=1}^{L_{fc}}2D_{j}^{1}\rho_{j}^{1}\right)$ *	$9.40\times 10^{8}\times N$
Algo.2	$\epsilon \times \frac{N}{\Omega }\times \left(\sum_{i=1}^{L_{\mathrm{conv}}}2Q_{i}^{2}R_{i}^{2}\varpi_{i}^{2}{\left(W_{i}^{2}\right)}^{2}\gamma_{i}^{2}+\sum_{j=1}^{L_{fc}}2D_{j}^{2}\rho_{j}^{2}\right)$ *	$2.00\times 10^{8}\times N$
Algo.3	$\epsilon \times N\times \left(2D^{3}\delta^{3}+3\xi^{3}{\left(\delta^{3}\right)}^{2}+2\xi^{3}\delta^{3}+2\delta^{3}\rho^{3}\right)$ *	$5.17\times 10^{6}\times N$
Algo.4	$\epsilon \times N\times \left(2D^{4}\delta^{4}+3\xi^{4}{\left(\delta^{4}\right)}^{2}+2\xi^{4}\delta^{4}+2\delta^{4}\rho^{4}\right)$ *	$2.53\times 10^{8}\times N$
Algo.5	$\epsilon \times N\times \left(2D^{5}\delta +3\xi^{5}{\left(\delta^{5}\right)}^{2}+2\xi^{5}\delta^{5}+2\delta^{5}\rho^{5}\right)$ *	$3.19\times 10^{8}\times N$

* $Q_{i}^{j}$, $R_{i}^{j}$, $\varpi_{i}^{j}$, $W_{i}^{j}$, $\gamma_{i}^{j}$, $D_{i}^{j}$, $\rho_{i}^{j}$(j=1,2) represent the parameters for Algo.1, Algo.2; $D^{j}$, $\delta^{j}$, $\xi^{j}$, $\rho^{j}$(j=3,4,5) represent the parameters for Algo.3, Algo.4, and Algo.5. (Algo.1: PE-MSRN; Algo.2: CNN; Algo.3: FCNet; Algo.4: FE-FCNet; Algo.5: PE-FCNet.)

**Table 4:** Simulation setting.

Parameter	Value
Frequency range	30 GHz
System bandwidth	200 MHz
Subcarrier spacing	60 kHz
Receiving antennas	64
The comb size of frequency domain $N_{\mathrm{SRS}}^{\mathrm{comb}}$	4
The number of time domain symbols $N_{\mathrm{SRS}}^{\mathrm{symbol}}$	2
The RB size of frequency domain $N_{\mathrm{SRS}}^{\mathrm{PRB}}$	262
The number of FFT points $N_{\mathrm{FFT}}$	4096

**Table 5:** Application scenarios and accuracy requirements.

Scenario ID	Application Scenario	Accuracy Requirement	Granularity
S1 (3D)	Outdoor highway traffic flow management	<10 m	50 m
S2 (2D/3D)	Tool tracking in smart factory	<4 m	10 m
S3 (2D/3D)	Indoor autonomous driving and asset localization	<30 cm	2 m

**Table 6:** The positioning accuracy indicators of the PE-MSRN algorithm with different training epochs ϵ.

ϵ	Mean Error (m)	90% Error (m)	Maximum Error (m)	<1 m Proportion (%)	<0.5 m Proportion (%)
20	1.0476	1.9507	3.2585	50.96%	10.76%
30	1.0338	1.8282	2.3731	50.88%	10.34%
50	0.7823	1.4606	2.2480	75.23%	22.94%
70	0.6676	1.2860	1.9783	84.57%	33.53%
80	0.6471	1.2568	1.9313	85.49%	36.70%
100	0.6178	1.2137	1.7694	88.07%	40.20%
120	0.6307	1.1993	1.8523	86.74%	38.70%

**Table 7:** Algorithm positioning accuracy indicators in the ablation experiments.

Model	Mean Error (m)	90% Error (m)	Maximum Error (m)	Minimum Error (m)	<1 m Proportion (%)	<0.5 m Proportion (%)
PE-MSRN	0.6178	1.0221	1.6098	0.0505	88.76%	39.43%
PE-MSN	0.6371	1.0570	2.2035	0.0447	87.04%	38.43%
PE-SRN	0.6365	1.0633	1.8312	0.0455	85.87%	37.54%
MSRN	0.8047	1.2825	2.0967	0.0660	72.19%	20.08%

**Table 8:** Positioning performance comparison for different algorithms in the S3 (2D) scenario.

Model	Mean Error (m)	90% Error (m)	Maximum Error (m)	Minimum Error (m)
Algo.1	0.5703	1.3788	2.7680	0.0077
Algo.2	0.6303	1.3823	2.3263	0.0216
Algo.3	1.3110	2.2006	3.6418	0.0456
Algo.4	1.2779	1.8368	2.7557	0.0390
Algo.5	0.7056	1.2519	2.0485	0.0235

Algo.1: PE-MSRN; Algo.2: CNN; Algo.3: FCNet; Algo.4: FE-FCNet; Algo.5: PE-FCNet.

**Table 9:** Positioning performance comparison for different algorithms in the S2 (2D) scenario.

Model	Mean Error (m)	90% Error (m)	Maximum Error (m)	Minimum Error (m)
Algo.1	1.6498	1.3788	4.2879	0.0547
Algo.2	1.9310	2.2006	5.6738	0.0456
Algo.3	4.3115	5.2281	6.2065	0.3481
Algo.4	3.0988	4.3062	6.1950	0.2680
Algo.5	2.1950	3.6075	6.1817	0.1736

Algo.1: PE-MSRN; Algo.2: CNN; Algo.3: FCNet; Algo.4: FE-FCNet; Algo.5: PE-FCNet.

**Table 10:** Positioning performance comparison for different algorithms in the S3 (3D) scenario.

Algorithm	Mean Error (m)	90% Error (m)	Maximum Error (m)	Minimum Error (m)	<1 m Proportion (%)	<0.5 m Proportion (%)
Algo.1	0.5728	1.0854	1.9056	0.0401	90.04%	45.94%
Algo.2	0.6326	1.1097	1.8968	0.0459	87.04%	37.93%
Algo.3	0.9120	1.3795	2.1230	0.0538	62.22%	12.84%
Algo.4	0.8659	1.3740	2.0737	0.0483	71.23%	22.10%
Algo.5	0.6434	0.7919	1.8043	0.0465	87.83%	32.38%

Algo.1: PE-MSRN; Algo.2: CNN; Algo.3: FCNet; Algo.4: FE-FCNet; Algo.5: PE-FCNet.

**Table 11:** Positioning performance comparison for different algorithms in the S2 (3D) scenario.

Model	Mean Error (m)	90% Error (m)	Maximum Error (m)	Minimum Error (m)
Algo.1	1.2915	2.2526	4.0681	0.0401
Algo.2	3.8905	6.3072	8.7825	0.3216
Algo.3	2.9760	4.3498	6.3629	0.2825
Algo.4	2.1842	3.7561	8.5306	0.1418
Algo.5	1.4253	2.3504	4.2653	0.0459

Algo.1: PE-MSRN; Algo.2: CNN; Algo.3: FCNet; Algo.4: FE-FCNet; Algo.5: PE-FCNet.

**Table 12:** Positioning performance comparison for different algorithms in the S1 (3D) scenario.

Model	Mean Error (m)	90% Error (m)	Maximum Error (m)	Minimum Error (m)
Algo.1	5.8188	9.2305	20.2470	0.5278
Algo.2	12.6442	17.3489	33.4930	1.0750
Algo.3	11.3788	17.3688	25.2940	1.1652
Algo.4	7.1863	11.5414	20.6883	0.6111
Algo.5	6.3544	9.8731	23.1124	0.7148

Algo.1: PE-MSRN; Algo.2: CNN; Algo.3: FCNet; Algo.4: FE-FCNet; Algo.5: PE-FCNet.

## Data Availability

The original contributions presented in this study are included in the article, and further inquiries can be directed to the corresponding authors.
